# SHP1 and SHP2 inhibition enhances the pro-differentiative effect of phorbol esters: an alternative approach against acute myeloid leukemia

**DOI:** 10.1186/s13046-019-1097-z

**Published:** 2019-02-14

**Authors:** Alejandro Pérez-Fernández, Guillermo López-Ruano, Rodrigo Prieto-Bermejo, Carla Ijurko, María Díez-Campelo, Fermín Sánchez-Guijo, Ángel Hernández-Hernández

**Affiliations:** 1 Department of Biochemistry and Molecular Biology, University of Salamanca, Edificio Departamental, Lab 122, Plaza Doctores de la Reina, S/N, P.O. 37007 Salamanca, Spain; 2 IBSAL, Institute for Biomedical Research of Salamanca, Virgen de la Vega Hospital, 10th floor, Paseo de San Vicente, 58-182, P.O. 37007 Salamanca, Spain; 3 Hematology Department, University Hospital of Salamanca, Paseo de San Vicente, 139, P.O. 37007 Salamanca, Spain

**Keywords:** SHP1, SHP2, Cell differentiation, Phorbol esters, Acute myeloid leukemia, Pro-differentiating therapy

## Abstract

**Background:**

The differentiation-based therapy for acute promyelocytic leukemia (APL) is an inspiring example for the search of novel strategies aimed at treatment of other subtypes of acute myeloid leukemia (AML). Thus, the discovery of new molecular players in cell differentiation becomes a paramount research area to achieve this goal. Here, the involvement of the protein tyrosine phosphatases SHP1 and SHP2 on leukemic cells differentiation is shown, along with the therapeutic possibilities of their targeting to enhance the differentiation induction effect of phorbol esters.

**Methods:**

The oxidation status and enzymatic activity of SHP1 and SHP2 during PMA-induced differentiation of HEL cells was evaluated. Additionally, the effects of RNAi-mediated downregulation of these phosphatases on cell differentiation was studied. Afterwards, the impact of chemical inhibition of SHP1 and SHP2 on differentiation both in the presence and absence of phorbol esters was tested. Finally, the anti-leukemic potential of phorbol esters and chemical inhibitors of SHP1 and SHP2 was addressed in several AML model cell lines, a xenograft mouse model and AML primary cells in vitro*.*

**Results:**

An increase of oxidation with a concomitant decrease of activity was observed for both phosphatases at the onset of PMA-induced differentiation. Consistently, silencing of these proteins favored the process, with an enhanced effect upon their simultaneous downregulation. Moreover, the proteins SRC and β-catenin were identified as downstream targets of SHP1 and SHP2 in this context. In agreement with these findings, chemical inhibition of the phosphatases promoted cell differentiation itself and enhanced the effect of phorbol esters. Interestingly, treatment with the phorbol ester prostratin and the dual inhibitor of SHP1 and SHP2 NSC87877 synergistically hampered the proliferation of AML cell lines, prolonged the survival of xenografted mice and reduced the clonogenic potential of AML primary cells.

**Conclusions:**

SHP1 and SHP2 are relevant mediators of differentiation in AML cells and their inhibition either alone or in combination with prostratin seems a promising differentiation-based therapeutic strategy against different subtypes of AML beyond APL.

**Electronic supplementary material:**

The online version of this article (10.1186/s13046-019-1097-z) contains supplementary material, which is available to authorized users.

## Background

Hematopoiesis is a paradigmatic differentiation process where a single cell type, the hematopoietic stem cell (HSC), gives rise to all mature blood lineages. A balanced collaboration of intrinsic and extrinsic factors regulates the decision between HSCs self-renewal and differentiation [1], thus ensuring a sustained blood production [2]. The alteration of this equilibrium can lead to a differentiation blockade and uncontrolled cell growth, what finally generates leukemogenesis [3].

In this regard, acute myeloid leukemia (AML), one of the most common types of leukemia, arises from either HSCs or more committed myeloid progenitors [4]. Despite the heterogeneous origin of leukemic clones, all AML patients share a hematopoietic differentiation arrest [5] that generates an accumulation of malignant blasts which outcompete mature healthy myeloid cells. The standard treatment consists of alternate cycles of cytarabine and anthracyclines followed by additional chemotherapy or transplantation [6]. However, the 5-year overall survival for adult AML patients is below 40% [7]. This highlights the need for developing new approaches that may help to revert such a fatal outcome.

The reduction of leukemic burden by overcoming the differentiation blockade has been pursued since the late 1970s [8]. The gold standard of this therapeutic strategy is the treatment of acute promyelocytic leukemia (APL) by combining all-*trans*-retinoic acid (ATRA) and arsenic trioxide. This has changed the outcome of patients from fatal to a virtual cure [9]. Due to the effectiveness of ATRA treatment on APL cells, its combination with other agents against non-APL AML has been tested in preclinical studies. Nevertheless, the rate of success seems to be dependent on molecular features yet to be fully understood [10]. Likewise, the discovery of novel pro-differentiating treatments applicable to non-APL AML seems to be an interesting approach, according to recent reports [11]. Regarding this issue, phorbol esters, like TPA (12-O-tetradecanoylphorbol-13-acetate) or PMA (phorbol-12-mysritate-13-acetate) are potent protein kinase C (PKC) agonists capable of inducing differentiation of leukemic cell lines [12] and primary cells [13]. Indeed, the effect of TPA has been already tested on patients suffering from myeloid leukemias [14, 15]. However, carcinogenic side-effects might have hampered their further development as therapeutic agents. Interestingly, a number of natural phorbol ester analogues like bryostatin 1 and prostratin (hereafter PRS) [16] seem not to be carcinogens, which reinforces their therapeutic feasibility. Therefore, a better understanding of the pathways involved in phorbol ester-mediated cell differentiation would be helpful for fine-tuning the development of alternative pro-differentiating approaches against leukemia.

As previously reviewed, reactive oxygen species (ROS) are important regulators of cell fate and differentiation processes, including hematopoiesis [17, 18]. ROS could modulate cell fate through the reversible oxidation of key signaling proteins, such as the oxidation-prone protein tyrosine phosphatases (PTPs) [19]. It has been shown before that the production of ROS through NADPH oxidases is required for phorbol ester-induced megakaryocytic differentiation in vitro [20], a fact that might be explained by the specific oxidation and inactivation of certain PTPs.

In the present work, the phorbol ester-mediated oxidation and inactivation of SHP1 and SHP2 was found. Both the silencing of these phosphatases and the use of NSC87877, enhanced cell differentiation. These results were exploited to develop a novel pro-differentiating therapeutic approach against non-APL AML. NSC87877 and the natural phorbol ester prostratin hampered the proliferation of several AML cell lines. Interestingly, the combination of both agents showed a synergistic effect. Moreover, the feasibility of this strategy was tested both in vivo and in bone marrow cells from non-APL AML patients. In summary, the results here obtained pave the way for a novel strategy based on the inhibition of SHP1 and SHP2 and the use of natural phorbol esters that may enlarge the therapeutic spectrum against AML.

## Methods

### Reagents

Cell culture reagents were from Biowest (Madrid, Spain). PMA (P8139) and pNPP (p-nitrophenyl phosphate, P4744) were from Sigma-Aldrich Spain. Stibogluconate sodium (HY-100595) was from MedChemExpress Europe (Sollentuna, Sweeden). Prostratin (sc-203422A) and NSC87877 (sc-204,139) were from Santa Cruz Biotechnology CA, USA. APC-conjugated CD41 (41A-100T) and CD61 (61A-100T) antibodies and Annexin V detection kit (ANXVKPE-100T) were from Immunostep (Salamanca, Spain). CD11b-APC antibody (130-191-241) and HSC-CFU Methylcellulose medium basic (130-091-275) and complete w/o EPO (130–091-227) were from Miltenyi Biotec (Madrid, Spain). Polyethylenimine (MW 25000, #23966–1) was from Polysciences, Inc. (Eppelheim, Germany). Antibodies and working dilutions used for western blot are detailed in Additional file 1: Table S1.

### Cell lines and primary samples

HEL (ACC-11), HL-60 (ACC-3) and NB4 (ACC-207) cells were purchased from DSMZ (Braunschweig, Germany). OCI-AML2 cells were purchased from ATCC. THP-1 cells were a gift from S. Lorenzo (University of Oviedo, Spain). All cell lines were grown in RPMI medium supplemented with 10% FBS, 100 U/ml penicillin, 100 U/ml streptomycin and 2 mM L-glutamine except OCI-AML2, which were grown in complete Alpha-MEM + FBS 20%.

Bone marrow mononuclear cells (BM-MNCs) were obtained as previously done [21] and cultured in RPMI medium supplemented with 10% FBS, 100 U/ml penicillin, 100 U/ml streptomycin and 2 mM L-glutamine. All cell lines were tested for *Mycoplasma spp.* contamination prior to use with PlasmoTest detection kit (InvivoGen, France, cat #rep-pt1).

### Detection of oxidized PTPs

The detection of oxidized PTPs was performed as described elsewhere [22]. Briefly, cells were lysed at room temperature for 20 min in previously degassed lysis buffer, (20 mM Tris pH 7.5, 10mM EDTA, 30 mM sodium pyrophosphate, 150 mM NaCl, 0.5% Triton X-100, 0.5% and sodium deoxycholate). The protein of interest was immunoprecipitated, and the sample was then treated with 50 mM iodoacetic acid to block reduced cysteines. The samples were then washed 3 times with 20 mM HEPES, and then treated with 100 mM DTT to reduce the oxidized Cys residues. Afterwards, they were washed again and treated with 100 μM pervanadate, which oxidizes the Cys residues that were not blocked by iodoacetic acid. Upon SDS-PAGE separation, the level of oxidation was monitored with an antibody against the oxidized PTP domain (Ox-PTP). The same blots were stripped and reprobed to detect the total level of the protein of interest.

### PTP enzyme activity

Cells were lysed 20 min on ice in previously degassed lysis buffer (25 mM HEPES pH 7.5, 150 mM NaCl, 1% IGEPAL, 10% glycerol, 1mM EDTA, 10 mM MgCl_2_, and 25 mM NaF). SHP1 and SHP2 were immunoprecipitated. Beads were resuspended in 50 mM HEPES pH 7.2, 150 mM NaCl, 50 mM KCl, 5 mM EDTA, and incubated at 37°C in the presence of 50 mM pNPP as a substrate. The enzyme activity was monitored by the increase of absorbance at 405 nm with respect to the unstimulated condition (t = 0 h).

### Immunoblotting

Immunoblotting and quantification of bands was performed as previously described [23]. GAPDH was used as loading control. Representative images of at least three different western blot experiments are shown.

### Lentiviral production for RNA interference

Sequences targeting the proteins of interest (see Additional file 1: Table S2) were designed and cloned into pLVTHM between MluI and ClaI sites. Lentivirus production and cell line transduction was done as described previously [21, 23, 24].

### Cell differentiation

Differentiation was monitored by flow cytometry analysis of the expression of the surface markers CD41 and CD61 and DNA content in HEL cells as before [20, 23] and by measuring the expression of CD11b in HL-60 cells [16]. Cell morphology was also assessed through observation of stained cytospins under a microscope.

### Cell viability, proliferation and clonogenic capacity

Cell viability was determined by Annexin V staining. Proliferation was followed by cell count in the presence of trypan blue and by MTT assays as before [21]. For colony-forming assays, cells were pre-treated for 48h with indicated drugs. Then, 500 HL-60 cells, 10,000 AML-derived BM-MNCs or 25,000 healthy donor-derived BM-MNCs were seeded per well in 0.5 ml of methylcellulose medium. Cells were grown at 37°C and 5% CO_2_ in an incubator and colonies were counted 7 days later for HL-60 cells and 14 days later for primary samples.

### Analysis of drug interactions

The interaction between the different drugs was analyzed by the median-effect method with CalcuSyn software (Biosoft, Cambridge, UK). For each combination, this algorithm calculates the combination index (CI), a quantitative parameter that defines synergy (CI < 1), additivity (CI = 1) or antagonism (CI > 1) between the compounds [25].

### In vivo xenograft mouse model

Mice were obtained from Charles River (Barcelona, Spain). Female 8-week-old NOD-SCID mice were irradiated with a 2.5 Gy single dose 24 h prior to cell transplant. HL-60 cells were maintained in exponential growth phase, washed twice with PBS and then resuspended in RPMI medium without FBS. Each animal was injected with 5·10^6^ HL-60 cells through the lateral tail veins. Cell engraftment was checked by flow cytometry. Five days after transplantation, treatment with the indicated agents was started. Stock solutions of drugs were diluted in sterile PBS and administered intraperitoneally (i.p.). Animals were daily monitored and treated every two days until humane endpoint, defined by scruffy fur, loss of activity and a loss of weight equal or greater than 25%, was reached. All animal protocols were approved by the University of Salamanca Bioethics Committee.

### Statistical analyses and data report

For two-group comparisons, Student’s *t* test was used. For multiple comparisons, either ANOVA (normal distributed data) or Kruskal-Wallis (not normal distributed data) tests were performed. For animal survival, a Log-Rank test was conducted. Excel, SPSS and R v3.4.4 were used as statistics software.

Results in bar graphs are given as mean values and their corresponding standard deviation. Numbers above bars indicate biological replicates. Lines above bar diagrams depict statistical significance of pairwise comparison between the bars located below the extremes when a multiple comparison test was performed. For all tests, differences were considered statistically significant when *p* < 0.05 (*), *p* < 0.01 (**), *p* < 0.001 (***).

## Results

### SHP1 and SHP2 are oxidized at the onset of HEL cells differentiation

**Fig. 1:**
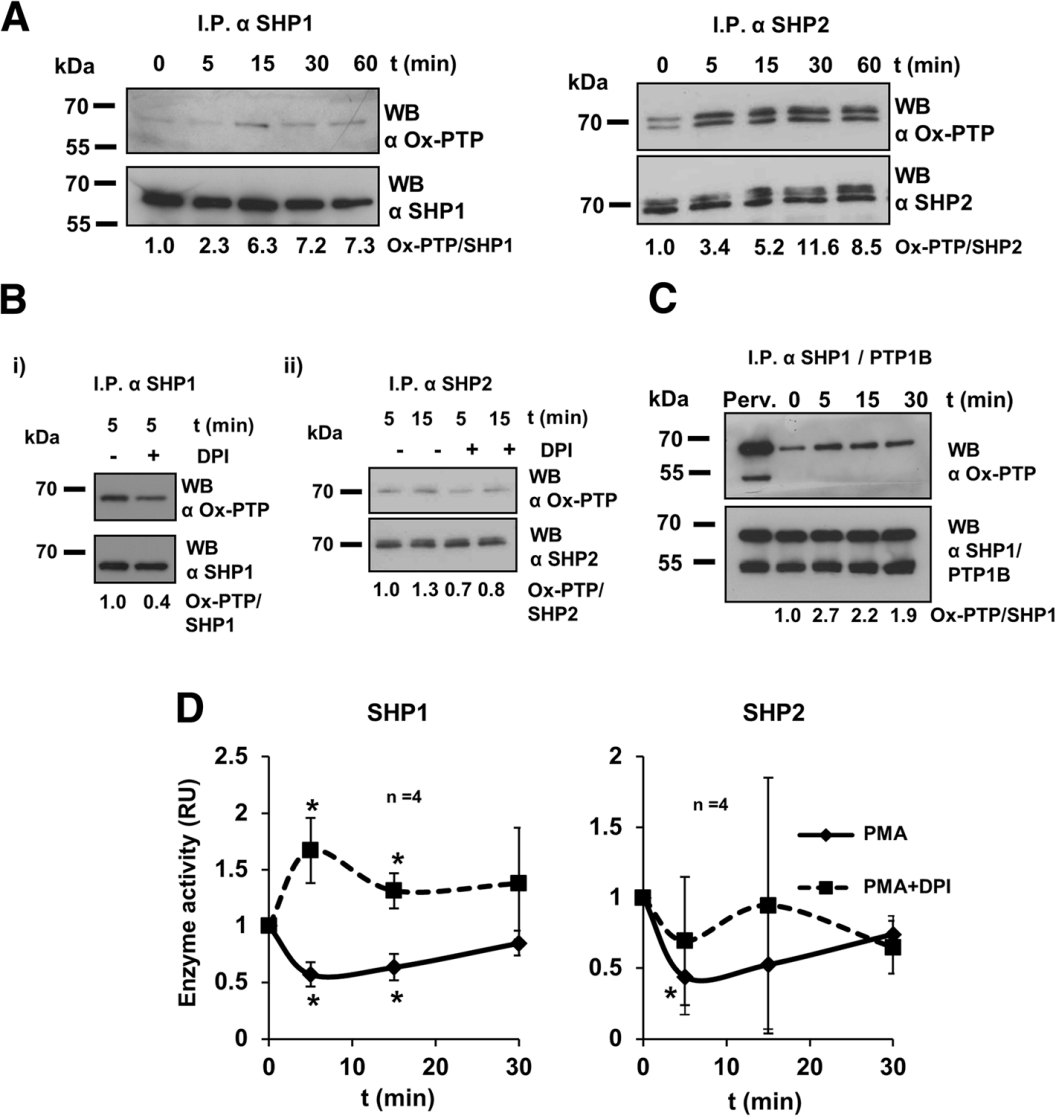
PMA induces a rapid oxidation and loss of activity of SHP1 and SHP2 phosphatases. **a**) Oxidized SHP1 and SHP2 upon stimulation of HEL cells with 20 nM PMA. **b**) Oxidation western blots of SHP1 and SHP2 after 20 nM PMA stimulation in the presence or absence of 5 μM DPI. **c**) Simultaneous monitoring of SHP1 and PTP1B oxidation upon 20 nM PMA treatment. First lane shows an extract from HEL cells treated with VO_4_^− 3^ before protein extraction as a positive control for oxidation. **d**) Phosphatase activity of SHP1 and SHP2 from HEL cells treated with 20 nM PMA either alone (solid lines) or with 5 μM DPI (dashed lines) at different time points. Asterisks (*) indicate statistical significance versus the unstimulated condition (t = 0 h)

As previously demonstrated, PMA-triggered cell differentiation relies on NADPH oxidase-generated ROS [20]. A role for ROS as second messengers through the oxidation of signaling proteins involved in differentiation, such as PTPs, seems a reasonable surmise. SHP1 and SHP2 are two intracellular PTPs involved in both normal hematopoiesis and leukemogenesis [26, 27]. Therefore, their redox status in response to PMA was evaluated. Both phosphatases were more oxidized upon activation of cell differentiation with PMA (Fig. 1a). The oxidation was prevented with diphenylene iodonium (DPI), a well-known inhibitor of NADPH oxidases (Fig. 1b). To test the specificity of SHP1 and SHP2 oxidation, the status of another PTP family member, PTP1B, was also examined. No oxidized PTP1B was detected in response to PMA, whereas oxidized SHP1 was clearly present in the same samples (Fig. 1c).

Reversible oxidation is one of the most important regulatory mechanisms of PTP activity [28, 29]. For this reason, the activity of SHP1 and SHP2 was measured. A decrease of activity was found after PMA stimulation, being more evident for SHP1 (Fig. 1d, solid lines). Co-treatment with DPI not only avoided the inactivation of SHP2, but also enhanced SHP1 activity, as previously reported [30] (Fig. 1d, dashed lines). This strongly suggests that NADPH oxidase-derived ROS production triggered by PMA would specifically oxidize and inactivate these phosphatases.

### Downregulation of SHP1 and SHP2, but not PTP1B, triggers cell differentiation

**Fig. 2:**
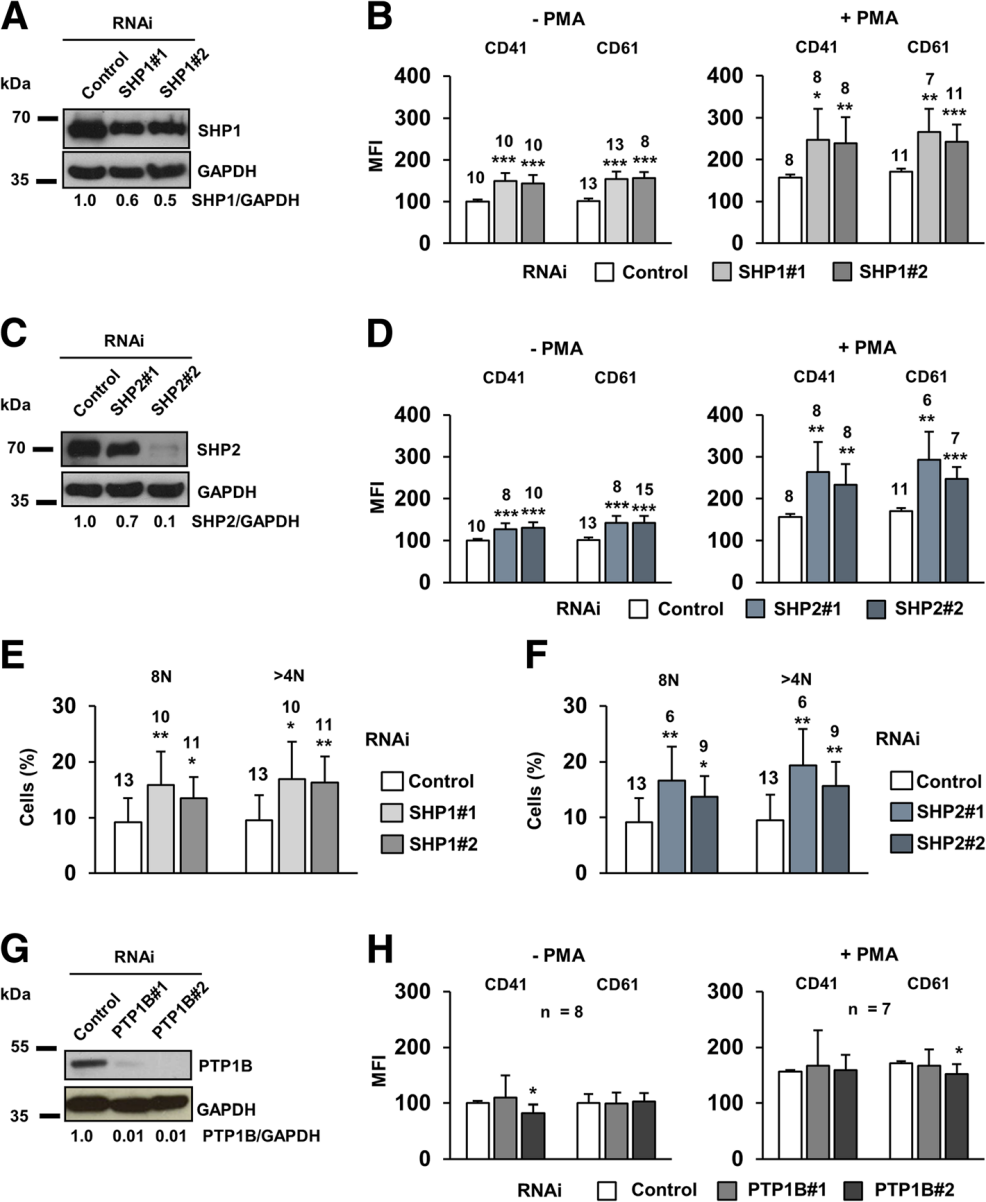
The silencing of SHP1 and SHP2 specifically enhances HEL cell differentiation. **a**) Downregulation of SHP1 with the RNAi sequences employed. **b**) Levels of CD41 and CD61 surface markers in SHP1-silenced cells untreated and stimulated with 20 nM PMA for 48 h. **c**) Downregulation of SHP2 with the RNAi sequences employed. **d**) Levels of CD41 and CD61 surface markers in SHP2-silenced cells untreated and stimulated for differentiation with 20 nM PMA for 48 h. **e**) Flow cytometry analysis of DNA content SHP1-silenced cells after 7 days of 20 nM PMA treatment. **f**) Flow cytometry analysis of DNA content SHP2-silenced cells after 7 days of 20 nM PMA treatment. **g**) Downregulation of PTP1B with the RNAi sequences employed. **h**) Levels of CD41 and CD61 surface markers in PTP1B-silenced cells untreated and stimulated for differentiation with 20 nM PMA for 48 h. All comparisons were made against control RNAi

SHP1 and SHP2 expression was reduced in HEL cells by RNAi (Fig. 2a, c) to explore the relevance of the rapid decrease of their activity in cell differentiation. HEL cells undertake differentiation upon stimulation with phorbol esters. This phenomenon is usually tracked through the expression increase of the epitopes CD41 (GpIIb/IIIa complex) and CD61 (GpIIIa) and the increase of cell ploidy [20, 23]. The complex GpIIb/IIIa is a receptor for fibrinogen and has a role in platelet aggregation, and the synthesis of both glycoproteins is augmented along megakaryocytic differentiation [31]. An increase of the differentiation markers CD41 and CD61 was observed concomitantly with the downregulation of SHP1 (Fig. 2b, left panel) and SHP2 (Fig. 2d, left panel). This phenomenon was also evident after PMA stimulation (Fig. 2b and d, right panels) and supported by a significant increase of cell ploidy (Fig. 2e and f). These data are consistent with the hypothesis that SHP1 and SHP2 inhibition by oxidation would facilitate PMA-induced cell differentiation. It must be highlighted that silencing PTP1B (Fig. 2g), which was not oxidized in our system (Fig. 1c), did not increase cell surface markers (Fig. 2h). These observations support the idea of a specific inhibition of SHP1 and SHP2 during HEL cells differentiation.

### Simultaneous downregulation of SHP1 and SHP2 renders a stronger differentiation than single silencing of each phosphatase

**Fig. 3:**
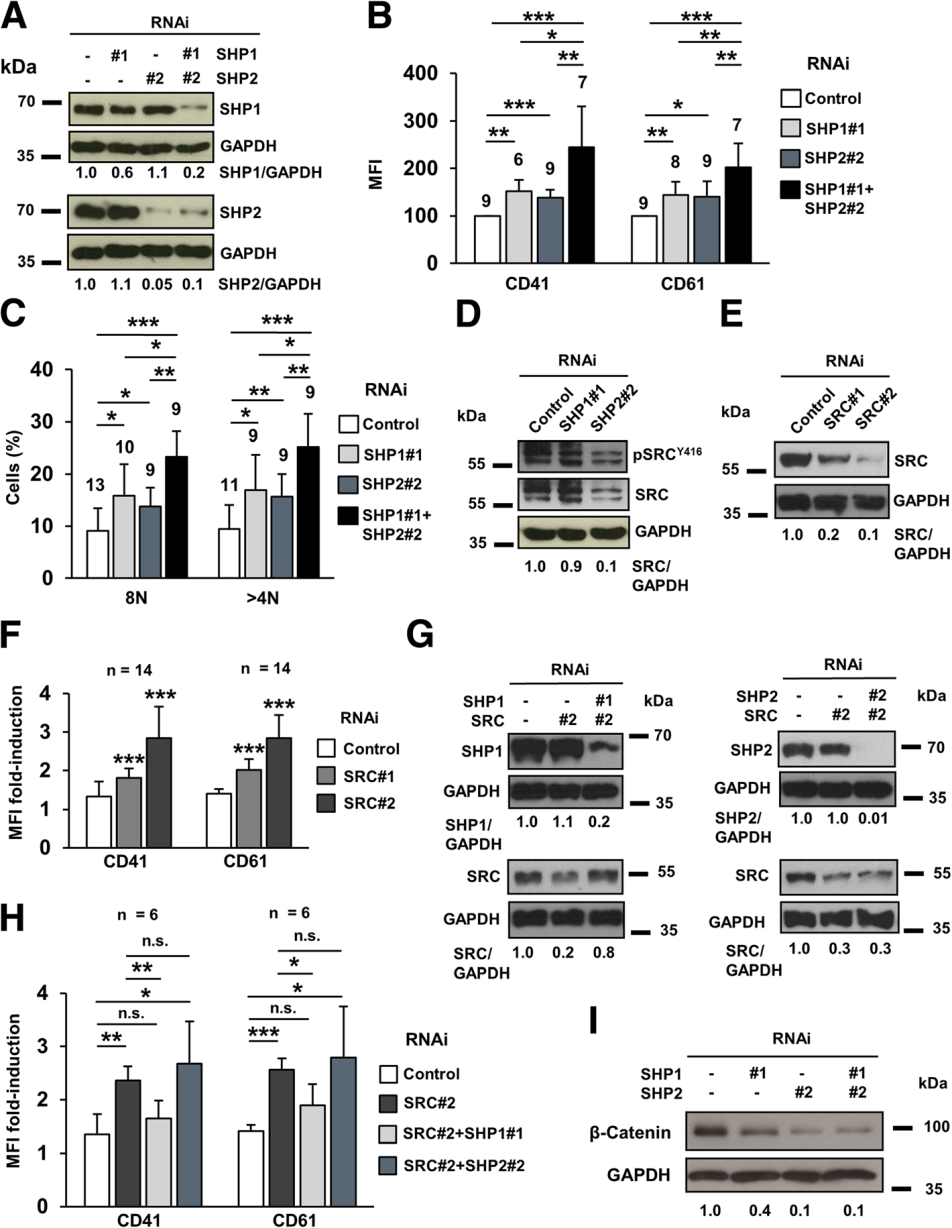
Simultaneous downregulation of SHP1 and SHP2 additively enhances cell differentiation through SRC and β-catenin. **a**) Levels of SHP1 and SHP2 when HEL cells were individually or simultaneously silenced for both proteins. **b**) Expression levels of differentiation markers in HEL cells individually and simultaneously silenced for both PTPs. **c**) DNA content of HEL cells individually and simultaneously silenced for both PTPs. **d**) Levels of total and phosphorylated SRC protein in SHP1- and SHP2- silenced HEL cells. **e**) SRC protein levels in HEL cells upon RNAi-mediated downregulation with two different sequences. **f**) Induction of the expression of the surface markers after 48 h treatment with 20 nM PMA in SRC-silenced HEL cells. Comparisons were performed against control RNAi. **g**) Simultaneous downregulation of SHP1 and SRC or SHP2 and SRC in HEL cells. **h**) Induction of the expression of the surface markers after 48 h treatment with 20 nM PMA in HEL cells simultaneously downregulated for SRC and either SHP1 or SHP2. **i**) Levels of β-catenin in HEL cells individually and simultaneously downregulated for SHP1 and SHP2

To elucidate whether SHP1 and SHP2 acted through the same or different pathways, both phosphatases were simultaneously downregulated in HEL cells (Fig. 3a). In this condition, differentiation markers (Fig. 3b) and cell ploidy (Fig. 3c) were significantly higher than in individually silenced cells. These additive effects allowed us to speculate that SHP1 and SHP2 could influence PMA-induced differentiation, at least partially, through different pathways.

### SHP2 silencing reduces SRC levels

SRC kinase is highly expressed in platelets and required for their activation after vascular injury [32]. SRC gain-of-function mutations induce defective megakaryopoiesis in humans [33]. In addition, SHP2 can mediate SRC activation [34]. Accordingly, both SRC and pSRC^Y418^ were significantly reduced in SHP2- but not in SHP1-silenced cells (Fig. 3d), thus placing this kinase as a downstream target of SHP2. Interestingly, SRC-downregulated cells (Fig. 3e) experienced a stronger induction of the differentiation markers upon PMA exposure (Fig. 3f).

Simultaneous silencing of SRC with SHP1 (Fig. 3g, left panel) and SHP2 (Fig. 3g, right panel) were performed to locate the kinase up/downstream of the phosphatases. Whereas SHP2 and SRC silencing did not alter the cell phenotype versus SRC-silenced cells, SHP1 silencing seemed to counteract the effects of SRC downregulation (Fig. 3h). These results strongly suggest that SRC acts downstream of SHP2 but not downstream of SHP1.

### SHP1 and SHP2 silencing induces the downregulation of β-catenin

The Wnt/β-catenin signaling pathway is involved in the regulation of HSCs homeostasis [24, 35]. β-catenin overexpression is commonly found in AML patients and is considered a poor prognostic factor [36]. In contrast, β-catenin downregulation has a pro-differentiating effect on leukemic cells [23]. This prompted us to study β-catenin levels in SHP1- and SHP2-silenced cells, being significantly reduced in both cases (Fig. 3i). This could explain the differentiation enhancement observed (Figs. 2and 3b-c). Therefore, the regulation of β-catenin stability could be a convergence point for SHP1- and SHP2-mediated control of cell differentiation.

### Chemical inhibition of SHP1 and SHP2 enhances the differentiation of HL-60 cells

**Fig. 4:**
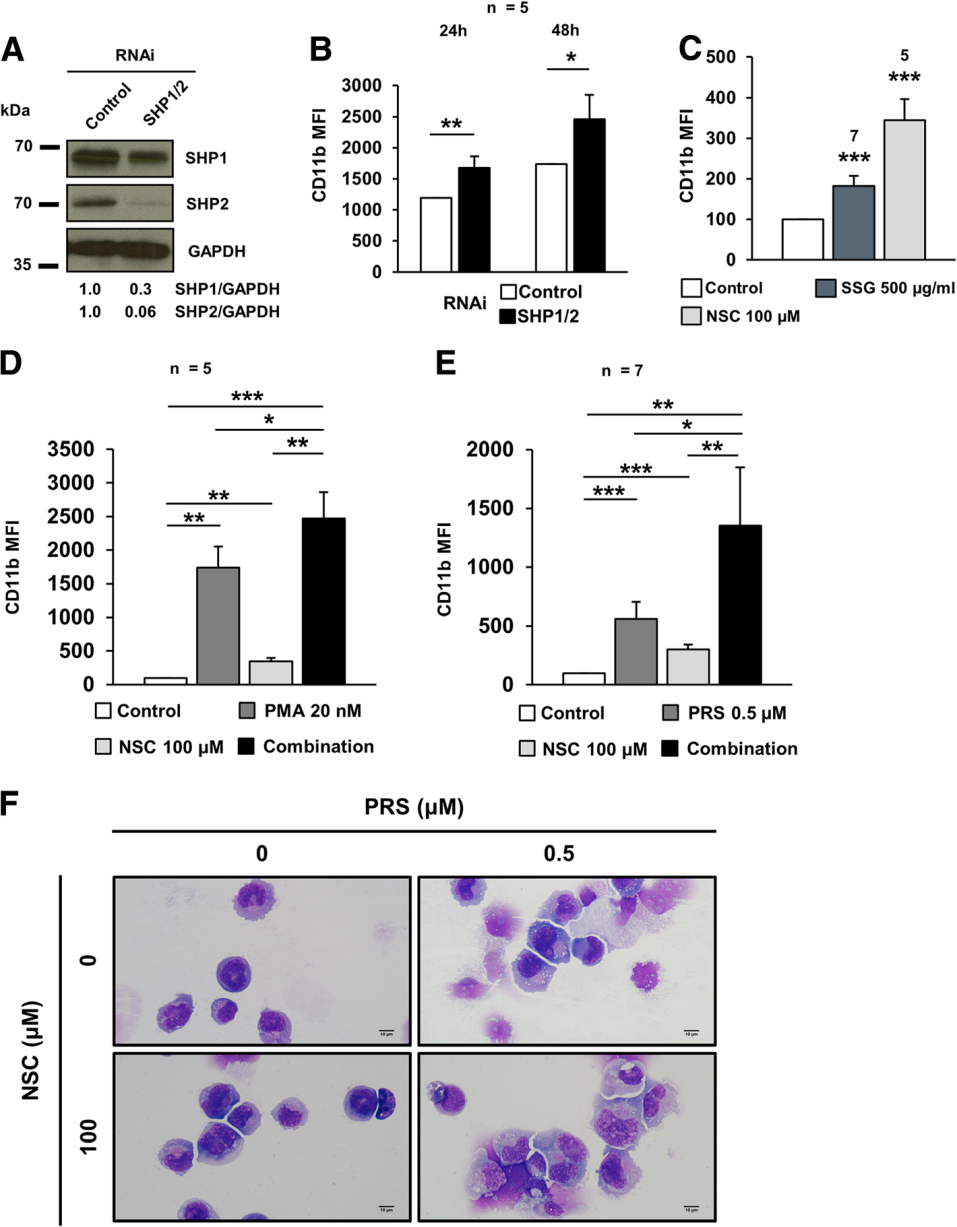
Chemical inhibition of SHP1 and SHP2 promotes differentiation in HL-60 cells. **a**) Silencing of both SHP1 and SHP2 in HL-60 cells. **b**) CD11b expression in SHP1- and SHP2-silenced HL-60 cells after treatment with PMA 20 nM during the indicated times. **c**) CD11b expression in HL-60 cells after 48 h treatment with SSG and NSC. **d**) CD11b expression in HL-60 cells after 48 h treatment with PMA, NSC and their combination. **e**) CD11b expression in HL-60 cells after 48 h treatment with PRS, NSC and their combination. **f**) Representative pictures of May-Grünwald-Giemsa stained cytospins of HL-60 cells after 48 h treatment with PRS, NSC and their combination. Scale bar: 10 μm

According to the observations made in HEL cells, the downregulation of SHP1 and SHP2 enhanced the levels of differentiation markers in other leukemic cell lines, such as K562 (our unpublished data) and HL-60. CD11b is upregulated upon phorbol ester stimulation of HL-60 cells [16] and is also a common marker to monitor differentiation of these cells. This antigen is the α subunit of the leukocyte integrin CD11b/CD18, involved in adhesion of activated neutrophils [37]. SHP1- and SHP2-silenced HL-60 cells (Fig. 4a) displayed an enhanced expression of CD11b over control cells upon PMA induction of differentiation (Fig. 4b). Both the successful pro-differentiating therapy currently used against APL [8] and the results shown previously were encouraging to test whether the chemical inhibition of SHP1 and SHP2 could trigger the differentiation of non-promyelocytic leukemic cells. Two different inhibitors of both SHP1 and SHP2, namely stibogluconate sodium (SSG) and NSC87877 (NSC), increased the expression of the differentiation markers CD41 and CD61 in HEL cells (Additional file 2: Figure S1) and CD11b in HL-60 cells (Fig. 4c). Moreover, NSC boosted the induction of CD11b expression by the phorbol esters PMA (Fig. 4d) and prostratin (Fig. 4e). These results were also confirmed at the morphological level, as shown by the reduced nucleus/cytoplasm ratio, the presence of more heterochromatic and kidney-shaped nuclei and the augmented cytosolic granulation in treated cells (Fig. 4f, Additional file 2: Figure S2). Altogether, the previous findings allow us to postulate the use of SHP1 and SHP2 inhibitors either alone or in combination with natural phorbol esters as a therapeutic strategy against non-APL AML.

### NSC87877 and prostratin act synergistically to prevent the proliferation of AML cell lines

**Fig. 5:**
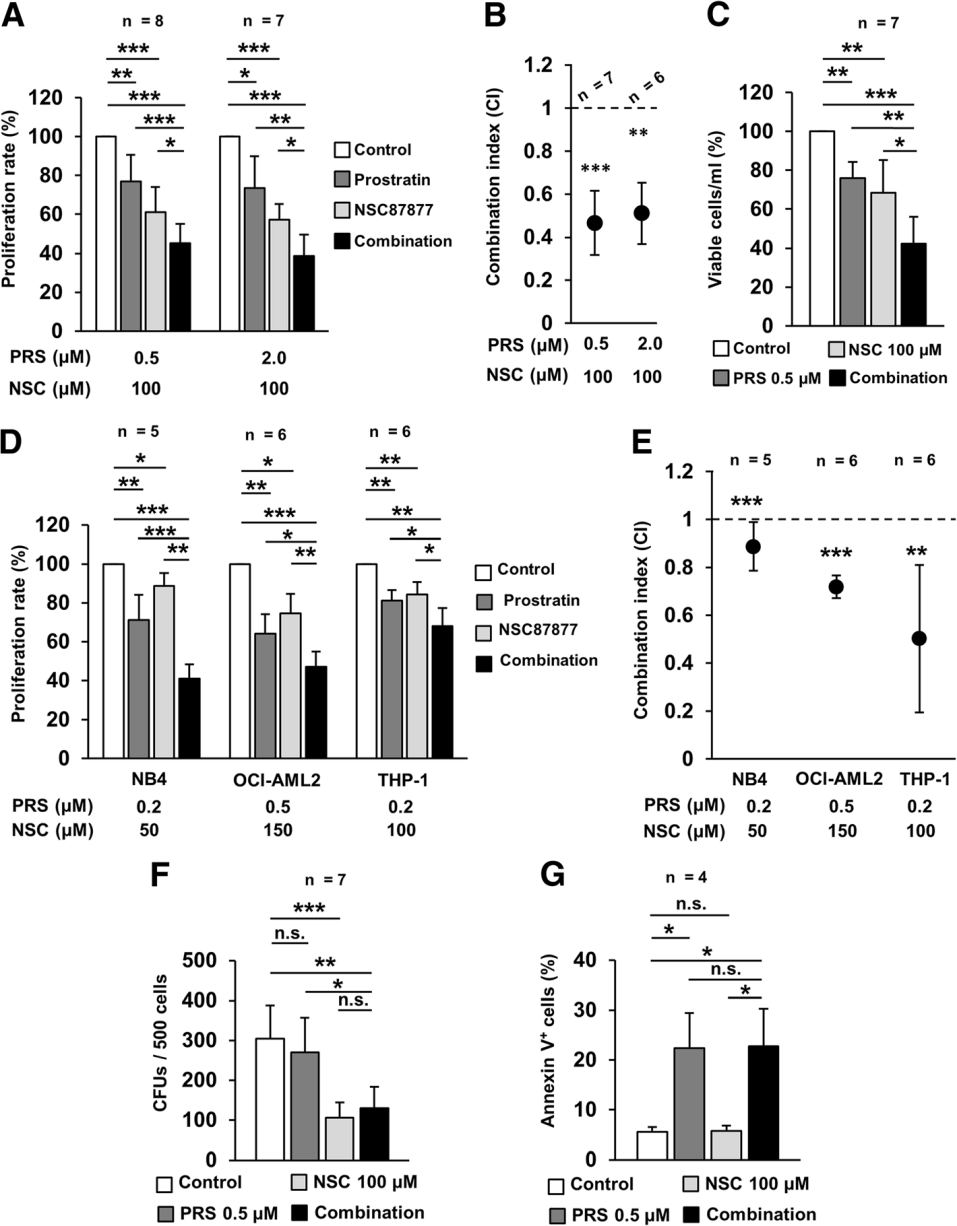
The combination of PRS and NSC synergistically prevents the proliferation of several AML cell lines. **a**) Effect of 48 h individual and combined treatments with PRS and NSC on HL-60 cells proliferation. **b**) Mean CI values for the drug combinations tested. Differences were tested between CI obtained values and 1. **c**) Percentage of viable HL-60 cells after 48 h of individual and combined treatments. **d**) Effect of 48 h treatment with the individual drugs and their combination on the proliferation rate of the specified cell lines. **e**) Mean CI values for the drug combinations tested in the different cell lines. Comparisons were made against the value 1. **f**) HL-60 colony numbers upon 48 h individual and combined treatment, drug removal, seeding and culture for 7 days. **g**) Percentage of apoptotic (Annexin V^+^) HL-60 cells after 48 treatment with the individual drugs and their combination

In light of the pro-differentiating effects described above, the feasibility of NSC87877, phorbol esters and their combination to slow down the growth of leukemia cells was evaluated. In this regard, PRS has been previously proposed as a non-tumorigenic alternative with therapeutic potential against leukemia [16]. Therefore, it was the only phorbol ester employed for subsequent experiments. Both NSC and PRS reduced HL-60 cells proliferation in a dose-dependent manner (Additional file 2: Figure S3A). Interestingly, the combined treatment with NSC and prostratin was more efficient than the single agents (Fig. 5a). Indeed, the combination indexes (CIs) indicated a synergistic impairment of cell proliferation for the drug combination (Fig. 5b). This enhanced inhibition of cell proliferation measured through MTT assays was further validated by a reduction of the cell count with trypan blue staining (Fig. 5c). This phenomenon held true in other AML model cell lines, both promyelocytic (NB4) and non-promyelocytic (OCI-AML2, THP-1, Fig. 5d-e), where different combinations were tested based on their responsiveness to the individual agents (Additional file 2: Figure S3B). In summary, these results support the feasibility of NSC, PRS and their combination as an alternative differentiation-based therapy against different AML subtypes beyond APL.

### Prostratin and NSC87877 contribute to preventing HL-60 cell growth through different mechanisms

The observed reduction in cell growth might be explained through two plausible phenomena: an increase in cell death and a reduction of the clonogenic ability. Both possibilities were therefore explored. After 48h exposure to the drugs and their combination, the clonogenic potential of HL-60 cells was evaluated. A mild but not significant reduction of the colony number was observed after PRS treatment, whereas exposure to NSC dramatically decreased HL-60 clonogenicity (Fig. 5f). This marked reduction of the colony number was also observed upon combined treatment, thus suggesting that NSC contributes to the synergistic effect observed through an impairment of clonal expansion. On the other hand, evaluation of cell death induction upon the same exposure time with Annexin V staining revealed an increase of apoptosis when cells were treated with both single PRS and its combination with NSC (Fig. 5g). These results would indicate a contribution to the synergistic effect by PRS through induction of apoptosis. All in all, NSC and PRS would be acting together to prevent proliferation of HL-60 cells through separate ways, the former by reducing clonogenicity and the latter by inducing cell death.

### Prostratin and NSC87877 effectively enhance survival of an animal model of leukemia

**Fig. 6:**
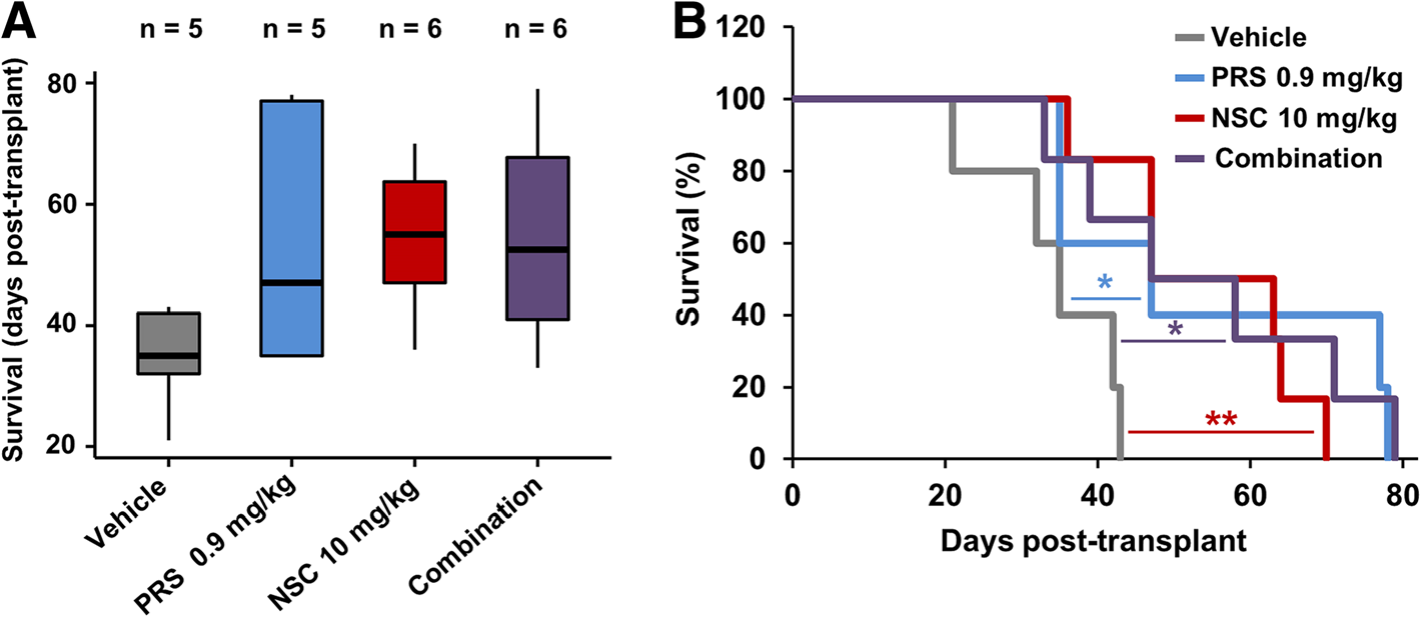
Treatment with prostratin and NSC87877 augments survival in an in vivo leukemia mouse model. **a**) Box plots showing the survival distribution of the animals treated with PRS, NSC and their combination. **b**) Kaplan-Meier plots showing the survival curves for immunodeficient mice transplanted with HL-60 cells and treated with PRS, NSC and their combination. Comparisons were made against control (vehicle) group

To further validate the findings shown with the in vitro approaches, a disperse xenograft with HL-60 cells in sublethally irradiated NOD-SCID mice was chosen as in vivo model. Survival of animals of each group was recorded and, as shown in Kaplan-Meier survival plots, both individual and combined treatments clearly enhanced animal survival with respect to control (Fig. 6). These results reinforce the possibility of employing NSC and PRS as a therapeutic approach against AML.

### The combination of prostratin and NSC87877 exerts an inhibitory effect on the clonogenic ability of AML primary samples

**Fig. 7:**
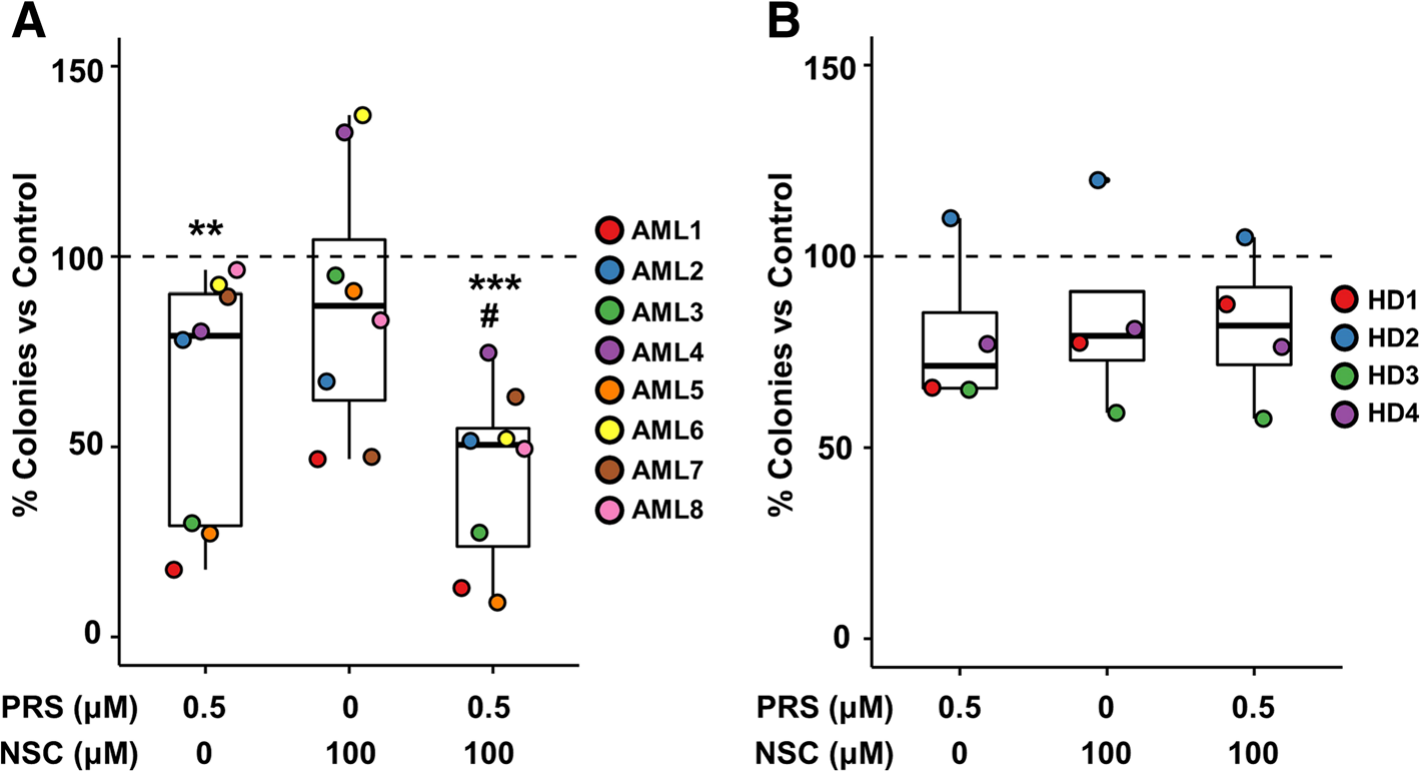
Effect of the individual and combined treatments with prostratin and NSC87877 on BM-MNCs*.* **a**) Relative colony numbers of BM-MNCs collected from AML patients upon treatment with drugs versus vehicle-treated cells. **b**) The same representation as in panel **a**) showing clonogenicity of healthy donor (HD)-derived BM-MNCs upon drug treatments. Values for each sample are individually plotted in different colors as indicated in the legend and box-plots are displayed as data summary. Asterisks (*) indicate statistical significance versus untreated cells and hashes (#) against NSC-treated cells

To address the clinical relevance of the drug combination tested on cell lines, 8 primary samples of newly diagnosed patients of non-APL AML (Table 1) and 4 samples from healthy donors (HD) were treated with the individual drugs and their combination at the synergistic doses in HL-60 cells. After 48 h exposure to the different treatments, drugs were withdrawn and the clonogenic potential was evaluated. A significant reduction in the colony-forming ability was triggered by both PRS alone and its combination with NSC (Fig. 7a). The combined treatment seemed more effective since a notorious decrease in the median percentage of colonies was observed versus single PRS treatment. In contrast, when healthy donor-derived BM-MNCs were exposed to the same drug doses, neither individual nor combined treatments showed a statistically significant reduction of the median percentage of colonies, with no enhanced effect of the co-treatment versus the individual drugs. Taken together, these results suggest some degree of specificity for the PRS + NSC co-treatment against primary acute myeloid leukemia cells versus healthy donor-derived bone marrow cells, thus raising the possibility for a therapeutic window in the clinical set up.

**Table 1:** Clinical features of patient samples used in the present study

ID	Age (years)	FAB subtype	Karyotype	Mutations
AML1	53	M0	46, XX, t(3;3)(q21;q21) [20]	*WT1* *IDH1*
AML2	59	M0	46, XY	N/D
AML3	57	M0	46, XY	N/A
AML4	52	M1	45, XX, −7 [15]	N/D
AML5	71	M0	46, XX, del (5q)(q13q35) [13] 47, XX_SL_, + 8 [2]	N/A
AML6	39	M4	46, XY	*FLT3*
AML7	50	Secondary AML	No metaphases	N/D
AML8	64	M5	No metaphases	*NPM1*

Legend: *N/A*: not available, *N/D*: not detected, *FAB*: French-American-British classification

## Discussion

The pro-differentiating therapy against APL [8] is a good example that encourages the search for analogous strategies applicable to other types of leukemia. Consequently, a better understanding of the driver mechanisms of cell differentiation could be helpful to unravel new molecular targets with therapeutic potential. This work has been developed based on two different phenomena: the ability of phorbol esters to induce cell differentiation [12], and the involvement of redox signaling in this process [20]. The first aspect has not gone unnoticed [14, 15], and recent preclinical reports have suggested the interesting possibility of using natural phorbol esters in cancer treatment [16, 38]. Regarding the second one, it must be stressed the fact that tumor cells display high levels of ROS as a hallmark. This could alter cellular signaling, thus leading to a higher proliferation [17, 18]. ROS production by NADPH oxidases has been related to patient-derived AML cells overproliferation [39]. Given the importance of ROS for cell differentiation, one could suggest a relationship between an altered redox signaling and the differentiation blockade found in AML. There is a direct link between redox signaling and phorbol ester-induced differentiation, since these compounds activate ROS production by NADPH oxidases [40].

With this background, some redox targets that could mediate phorbol ester-induced cell differentiation were investigated. Specific oxidation and inhibition of two intracellular phosphatases (SHP1 and SHP2) was shown as required to trigger cell differentiation. These results agree with other reports showing the oxidation of SHP1 [41] and SHP2 [42] by NADPH oxidase-produced ROS. Indeed, the activity decrease of SHP1 upon PMA treatment was not only rescued when cells were co-incubated with DPI but raised versus untreated condition. This event was previously reported in primary cells of hairy cell leukemia, where SHP1 was found in cell membrane in close proximity to Nox5, which may oxidize and inactivate the phosphatase by generation of ROS [30]. Our findings would be consistent with a similar mechanism operating in our system, given the involvement of Nox family members in HEL cells differentiation [20]. Moreover, enhanced cell differentiation by inactivation of both phosphatases was confirmed by RNAi-mediated silencing. It must be highlighted the confluent effect on cell differentiation observed with these approaches, considering that SHP1 and SHP2 usually exert opposite roles in signaling pathways [43, 44]. This prompted us to study the pathways affected by the inactivation of these phosphatases.

The regulation of SRC activity by tyrosine phosphorylation is a well-known event, and the involvement of different PTPs, including SHP1 and SHP2, in this process, has been suggested [45]. Less attention has been paid to the levels of SRC, despite that the upregulation of its stability and synthesis promotes breast cancer metastasis [46]. In that report the authors showed that SRC can be specifically degraded by calpain. Here, a reduction in SRC levels upon SHP2 silencing was found, thus suggesting a positive regulation of SRC stability by SHP2. Interestingly, a recent report proposed a SHP2-mediated negative regulation of calpain activity [47]. This allows us to speculate that SHP2 could regulate SRC stability by modulating calpain activity. Also, the inhibition of SRC can facilitate leukemic cells differentiation [48], a fact consistent with the stronger induction of differentiation in the SRC-silenced cells shown in this work.

Constitutive activation of β-catenin can contribute to tumorigenesis and, in particular, to AML development [49]. In agreement with these findings, inhibition of Wnt/β-catenin signaling pathway has been recently pointed as a therapeutic approach against FLT3-mutated AML [50]. It has been also shown before that leukemic cell differentiation is triggered by β-catenin downregulation [23]. Although SHP1-induced β-catenin degradation has been reported [51], a notorious decrease of β-catenin upon both SHP1 and SHP2 silencing was observed here, a fact that could explain the induction of cell differentiation under this same conditions.

Chemical inhibitors of SHP1 and SHP2 induced cell differentiation and enhanced the effect of phorbol esters. Therefore, the possibility of a therapeutic use of these compounds against AML cells was next analyzed. Importantly, NSC87877, the natural phorbol ester prostratin, and their combination were effective against a panel of different AML cell lines, thus supporting a wide therapeutic potential against this disease.

As a proof of concept of the translatability of this strategy into the clinic, its effect on a HL-60 xenograft mouse model and non-APL AML bone marrow cells was evaluated. The first approach showed the efficacy of both PRS and NSC to increase animal survival. Moreover, the analysis of the clonogenic ability of bone marrow cells from AML patients suggested the benefit of using the combination of PRS and NSC. It is noteworthy the fact that samples AML1, AML3 and AML5 were considerably more responsive to PRS and PRS + NSC than the others. All of them belong to FAB subtype M0 (Table 1), greatly associated with a poor prognosis [52]. This is a very relevant finding, given the urgent need for finding new therapeutic approaches against this intrinsically chemoresistant rare entity. Interestingly, neither significant effect on healthy donor-derived cells nor enhancement of the action of individual drugs upon co-treatment was found.

## Conclusion

The treatment of APL with ATRA and arsenic trioxide has shown how overcoming the differentiation blockade associated to leukemogenesis leads to a dramatic decrease of leukemic burden. A better understanding of leukemic cell differentiation would allow for the design of analogous therapies applicable to other types of AML. The possibility of using natural phorbol esters, such as prostratin, for cancer treatment, prompted us to analyze the mechanism of phorbol ester-induced leukemic cell differentiation. A link between the induction of such differentiation and the specific oxidation and inhibition of two intracellular phosphatases, SHP1 and SHP2, is shown here. The dual inhibition of these enzymes in combination with prostratin induces leukemic cell differentiation and inhibits cell proliferation in a synergistic manner. The effects in animal survival and colony-forming ability of bone marrow cells from patients support the notion that this strategy would be a feasible approach for a future non-APL AML treatment.

## Additional files

Additional file 1: **Table S1.** Primary antibodies and working dilutions used for immunoblotting. **Table S2.** RNAi sequences for downregulation of the proteins studied in this work. (DOCX 16 kb)

Additional file 2: **Figure S1.** Chemical inhibition of SHP1 and SHP2 favors the differentiation of HEL cells. Levels of CD41 and CD61 surface markers in HEL cells 48 h after 4h incubation with the indicated inhibitors of SHP1 and SHP2. **Figure S2.** Chemical inhibition of SHP1 and SHP2 potentiates morphological changes of PMA in HL-60 cells. Representative pictures of May-Grünwald-Giemsa stained cytospins of HL-60 cells after 48 h treatment with PRS, NSC and their combination. Scale bar: 10 μm. **Figure S3.** AML cell lines are differentially responsive to PRS and NSC. **A)** Dose-response curves of HL-60 cells treated with PRS and NSC during 48 h. **B)** Dose-response curves of AML cell lines different from HL-60 treated with PMA and NSC during 48 h. (DOCX 509 kb)

## Abbreviations

AML: Acute myeloid leukemia
APL: Acute promyelocytic leukemia
ATRA: All-*trans*-retinoic acid
BM-MNCs: Bone marrow mononuclear cells
CFU: Colony-forming unit
DPI: Diphenyleneiodonium
FBS: Fetal Bovine Serum
FLT3: FMS-like tyrosine kinase receptor 3
GAPDH: Glyceraldehyde-3-phosphate dehydrogenase
HSC: Hematopoietic stem cell
MTT: 3-(4, 5-dimethylthiazolyl-2)-2, 5-diphenyltetrazolium bromide
NOD-SCID: Non-obese diabetic-severe combined immunodeficient
PKC: Protein kinase C
PMA: Phorbol-12-mysritate-13-acetate
pNPP: p-nitrophenyl phosphate
PRS: Prostratin
PTP: Protein tyrosine phosphatase
PTP1B: Protein tyrosine phosphatase 1B
ROS: Reactive oxygen species
SHP1: Src homology 2 domain-containing protein tyrosine phosphatase 1
SHP2: Src homology 2 domain-containing protein tyrosine phosphatase 2
SRC: SRC proto-oncogene, non-receptor tyrosine kinase
SSG: Stibogluconate sodium
TPA: 12-O-tetradecanoylphorbol-13-acetate
