# Supplementary material for: SHP1 and SHP2 inhibition enhances the pro-differentiative effect of phorbol esters: an alternative approach against acute myeloid leukemia
Source: J Exp Clin Cancer Res. 2019 Feb 14;38:80. doi: 10.1186/s13046-019-1097-z (PMC6376690; doi:10.1186/s13046-019-1097-z)
Supplement: Supplementary file 1 — Table S1. Primary antibodies and working dilutions used for immunoblotting. Table S2. RNAi sequences for downregulation of the proteins studied in this work. (DOCX 16 kb) [file 13046_2019_1097_MOESM1_ESM.docx]

**Additional file 1:Table S1.** Primary antibodies and working dilutions used for immunoblotting

| **Target** | **Supplier** | **Reference (Clone)** | **Working dilution** |
| --- | --- | --- | --- |
| GAPDH | ThermoFisher Scientific | AM4300 (6C5) | 1:40000 |
| OxPTP | R&D Systems | MAB2844 | 1:500 |
| pSRC (Y418) | Abcam | ab4816 | 1:2000 |
| PTP1B | Santa Cruz Biotechnology | sc-14021 (H-135) | 1:2000 |
| SHP1 | Santa Cruz Biotechnology | sc-287 (C-19) | 1:2000 |
| SHP1 | BD Transduction Laboratories | #610126 (52/PTP1C/SHP1) | 1:1000 |
| SHP2 | Santa Cruz Biotechnology | sc-280 (C-18) | 1:2000 |
| SHP2 | BD Transduction Laboratories | #610622 (79/PTP1D/SHP2) | 1:2000 |
| SRC | Santa Cruz Biotechnology | sc- 8056 (B-12) | 1:2000 |
| SRC | Cell Signalling Technology | #2108S | 1:1000 |
| β-catenin | BD Transduction Laboratories | #610153 (14/Beta-Catenin) | 1:1000 |

**Table S2.** RNAi sequences for downregulation of the proteins studied in this work.

| **Target** | **Sequence ID** | **Nucleotide sequence** |
| --- | --- | --- |
| Firefly luciferase | Cont | 5'-CTGACGCGGAATACTTCGA-3' |
| SHP1 | SHP1#1 | 5'-GGAACAAATGCGTCCCATA-3' |
|  | SHP1#2 | 5'-TGACAGAGCTGGTGGAGTA-3' |
| SHP2 | SHP2#1 | 5'-GGATGGTGTTCCAAGAAAA-3' |
|  | SHP2#2 | 5'-TGACAGATCTTGTGGAACA-3' |
| SRC | SRC#1 | 5'-TCAAGTGCATTAAGAACGA-3' |
|  | SRC#2 | 5'-TCAAGTGCATTAAGAACGA-3' |
| PTP1B | PTP1B#1 | 5'-AGAAAGTGCTGTTAGAAAT-3' |
|  | PTP1B#2 | 5'-CTGAAGATATCAAGTCATA-3' |
|  |  |  |
