# Supplementary material for: SHP1 and SHP2 inhibition enhances the pro-differentiative effect of phorbol esters: an alternative approach against acute myeloid leukemia
Source: J Exp Clin Cancer Res. 2019 Feb 14;38:80. doi: 10.1186/s13046-019-1097-z (PMC6376690; doi:10.1186/s13046-019-1097-z)
Supplement: Supplementary file 2 — Figure S1. Chemical inhibition of SHP1 and SHP2 favors the differentiation of HEL cells. Levels of CD41 and CD61 surface markers in HEL cells 48 h after 4h incubation with the indicated inhibitors of SHP1 and SHP2. Figure S2. Chemical inhibition of SHP1 and SHP2 potentiates morphological changes of PMA in HL-60 cells. Representative pictures of May-Grünwald-Giemsa stained cytospins of HL-60 cells after 48 h treatment with PRS, NSC and their combination. Scale bar: 10 μm. Figure S3. AML cell lines are differentially responsive to PRS and NSC. A) Dose-response curves of HL-60 cells treated with PRS and NSC during 48 h. B) Dose-response curves of AML cell lines different from HL-60 treated with PMA and NSC during 48 h. (DOCX 509 kb) [file 13046_2019_1097_MOESM2_ESM.docx]

**
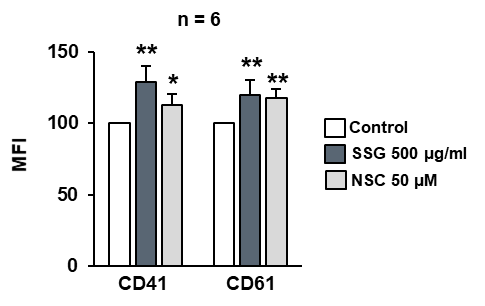
Additional file 2: Figure S1**

**Chemical inhibition of SHP1 and SHP2 favors the differentiation of HEL cells.** Levels of CD41 and CD61 surface markers in HEL cells 48 h after 4h incubation with the indicated inhibitors of SHP1 and SHP2.


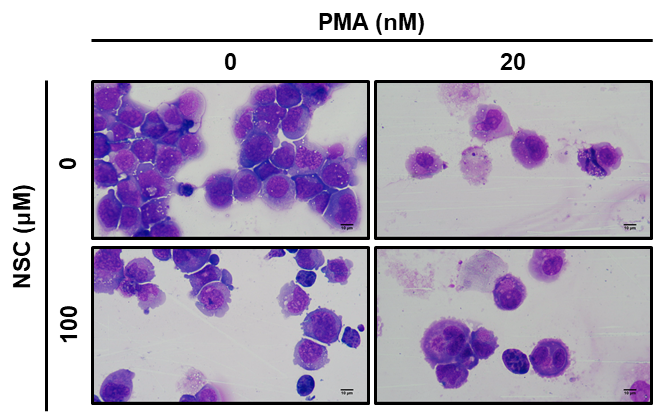
**Figure S2**

**Chemical inhibition of SHP1 and SHP2 potentiates morphological changes of PMA in HL-60 cells**. Representative pictures of May-Grünwald-Giemsa stained cytospins of HL-60 cells after 48 h treatment with PRS, NSC and their combination. Scale bar: 10 µm.

**
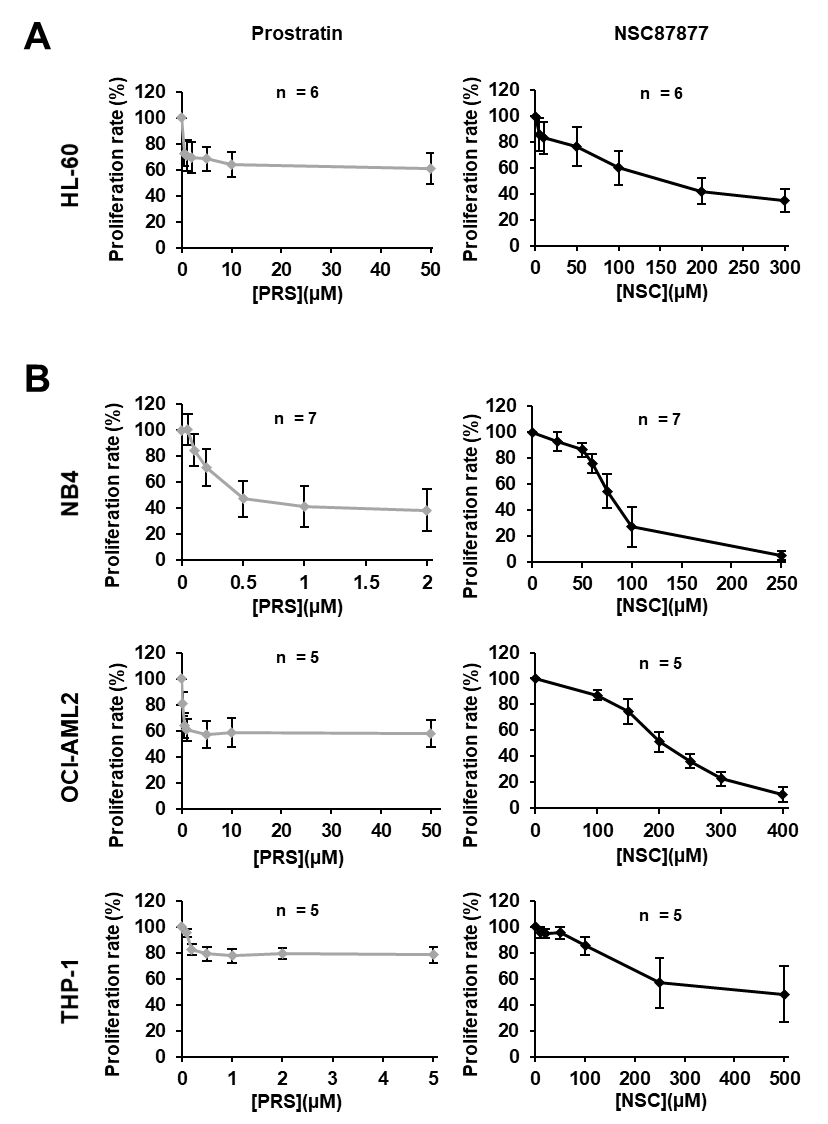
Figure S3**

**AML cell lines are differentially responsive to PRS and NSC. A)** Dose-response curves of HL-60 cells treated with PRS and NSC during 48 h. **B)** Dose-response curves of AML cell lines different from HL-60 treated with PMA and NSC during 48 h.
